# Reduction of Radiation Exposure in Atrioventricular Nodal Reentrant Tachycardia Ablations Using an Electroanatomical Mapping System With Fluoroscopy Integration Module

**DOI:** 10.3389/fcvm.2021.728422

**Published:** 2021-10-20

**Authors:** Christian Blockhaus, Jan-Erik Gülker, Alexander Bufe, Melchior Seyfarth, Buelent Koektuerk, Dong-In Shin

**Affiliations:** ^1^Department of Cardiology, Heart Centre Niederrhein, Helios Clinic Krefeld, Krefeld, Germany; ^2^Witten-Herdecke University, Witten, Germany; ^3^Department of Cardiology, Petrus Hospital, Wuppertal, Germany; ^4^Department of Cardiology, University Hospital Helios Wuppertal, Wuppertal, Germany

**Keywords:** fluoroscopy, electroanatomical mapping system, AVNRT, dose area product, ablation, electrophysiology

## Abstract

**Introduction:** Atrioventricular nodal reentrant tachycardia (AVNRT) is a common supraventricular tachycardia. Current guidelines recommend electrophysiology study (EPS) and ablation, which have been proven to show high success rates with very low complication rates. Usually, ablation of AVNRT is performed conventionally using only fluoroscopy. Electroanatomical mapping systems (EMS) are widely used in complex arrhythmias. One of their advantages is their potential in decreasing the need of fluoroscopy time (FT). In this study we analyzed patients undergoing either conventional AVNRT ablation or by using an EMS with a fluoroscopy integrating system (FIS).

**Materials and Methods:** We included 119 patients who underwent AVNRT ablation in our study. Eighty-nine patients were ablated conventionally using only fluoroscopy, 30 patients were ablated using EMS + FIS.

**Results:** We found that the use of EMS + FIS led to a significant reduction of FT (449.90 ± 217.21 vs. 136.93 ± 109.28 sec., *p* < 0.001) and dose-area-product (DAP, 268.27 ± 265.20 vs. 41.07 ± 27.89 μGym^2^, *p* < 0.001) without affecting the procedure time (PT, 66.55 ± 13.3 vs. 67.33 ± 13.81 min, *p* = 0.783). Furthermore, we found no significance with regard to complications.

**Conclusion:** The use of EMS+FIS is safe and feasible. It leads to a significant reduction of both FT and DAP without affecting PT and safety. Hence, EMS + FIS is beneficial for both the operator and the patients by reducing the radiation exposure.

## Introduction

Atrioventricular nodal reentrant tachycardia (AVNRT) is a common supraventricular tachycardia (SVT). It appears more often in women than in men. Furthermore, it is mostly seen in younger people without known heart disease (1). The ECG shows a narrow complex tachycardia with heart rate of about 170–210 beats per minute. Clinically, the patients often suffer from neck pounding and have visible pulsations in the neck, colloquially referred to as a “frog-sign” (2). Electrophysiology study (EPS) for diagnosis and ablation in AVNRT is an established treatment and recommended by current guidelines (class 1 recommendation) with a high success and a low complication rate (<1%) also leading to an increase in patients' quality of life (1, 3–5). During EPS the patient and the operator as well as the staff are exposed to radiation. The correlation of radiation exposure and the development of potential malignancies is well-known (6, 7). Operators in electrophysiology laboratories and catheterization labs have an elevated risk of developing malignancies (8). There is rising awareness that reduction of fluoroscopy is both beneficial for the patient, the operator and the laboratory personnel and many studies focus on the aim of zero-fluoroscopy ablations. Helpful manuals to reduce radiation in the electrophysiology laboratory have been published (9). Three-dimensional electroanatomic mapping systems (EMS) have been developed and are widely used in complex arrhythmias. They enable the operator to reconstruct a three-dimensional surface of a cardiac chamber, they show real-time position of catheters and they provide local electrophysiological information (10). EMS also have the potential to significantly reduce radiation. Their use and benefit have been widely shown. Disadvantages are higher costs and required operator training. Therefore, in many clinics AVNRT is mostly ablated conventionally using only fluoroscopy. The use of EMS with a fluoroscopy integrating system (FIS) has recently been introduced and several studies report on its fluoroscopy saving potential compared to EMS alone (11, 12). To use the FIS, a registration plate, attached to the location pad, and a software update are needed. After registration the module offers real-time visualization of catheters against a background of stored fluoroscopy images or even cines. Here, we report on our experiences with this EMS/FIS system in the ablation of AVNRT with respect to procedure time (PT), fluoroscopy time (FT), dose-area-product (DAP) and safety.

## Materials and Methods

We retrospectively analyzed 119 consecutive patients who underwent EPS with ablation of AVNRT in our clinic between 2018 and 2019. We performed 89 conventional ablations using only fluoroscopy and 30 ablations using an EMS (CARTO3, Biosense Webster, Diamond Bar, CA, USA) with a FIS (CARTO UNIVU™ module, Biosense Webster).

Written informed consent was obtained from all patients before the procedure. All ablations were performed under sedation with intravenous midazolam and propofol by an experienced operator. If oral anticoagulation was taken it was withheld for the day of the procedure. In conventional ablations, two diagnostic catheters (Biosense Webster) were inserted after femoral vein puncture via 6F sheaths and placed in the right ventricular apex (RVA) and the coronary sinus (CS). Ventricular and atrial programmed stimulation was performed to induce AVNRT with one or two extra beats, with different basic cycle lengths and burst stimulation from the atrium. In case of non-inducibility isoproterenol was given intravenously until the heart rate accelerated by at least 20% and the stimulation maneuvers were repeated. The stimulation maneuvers have been previously described elsewhere (13).

Indication for ablation was either the induction of AVNRT or the proof of an AH interval jump (minimum 50 ms) phenomenon plus echo beat(s) as a sign of dual AV node physiology in addition to a typical clinical presentation and prior ECG documentation of the tachycardia. After diagnosis of AVNRT an ablation catheter (Celsius, Biosense Webster, d- or f-curve) was inserted via an 8F sheath, HIS region was determined visually by fluoroscopy and local electrocardiogram and radiofrequency ablation was performed with a maximum of 40 Watts for 60 s at the posteroseptal region of the tricuspid annulus at sites with local electrophysiology signals compatible with the slow AV nodal pathway.

**Figure 1:**
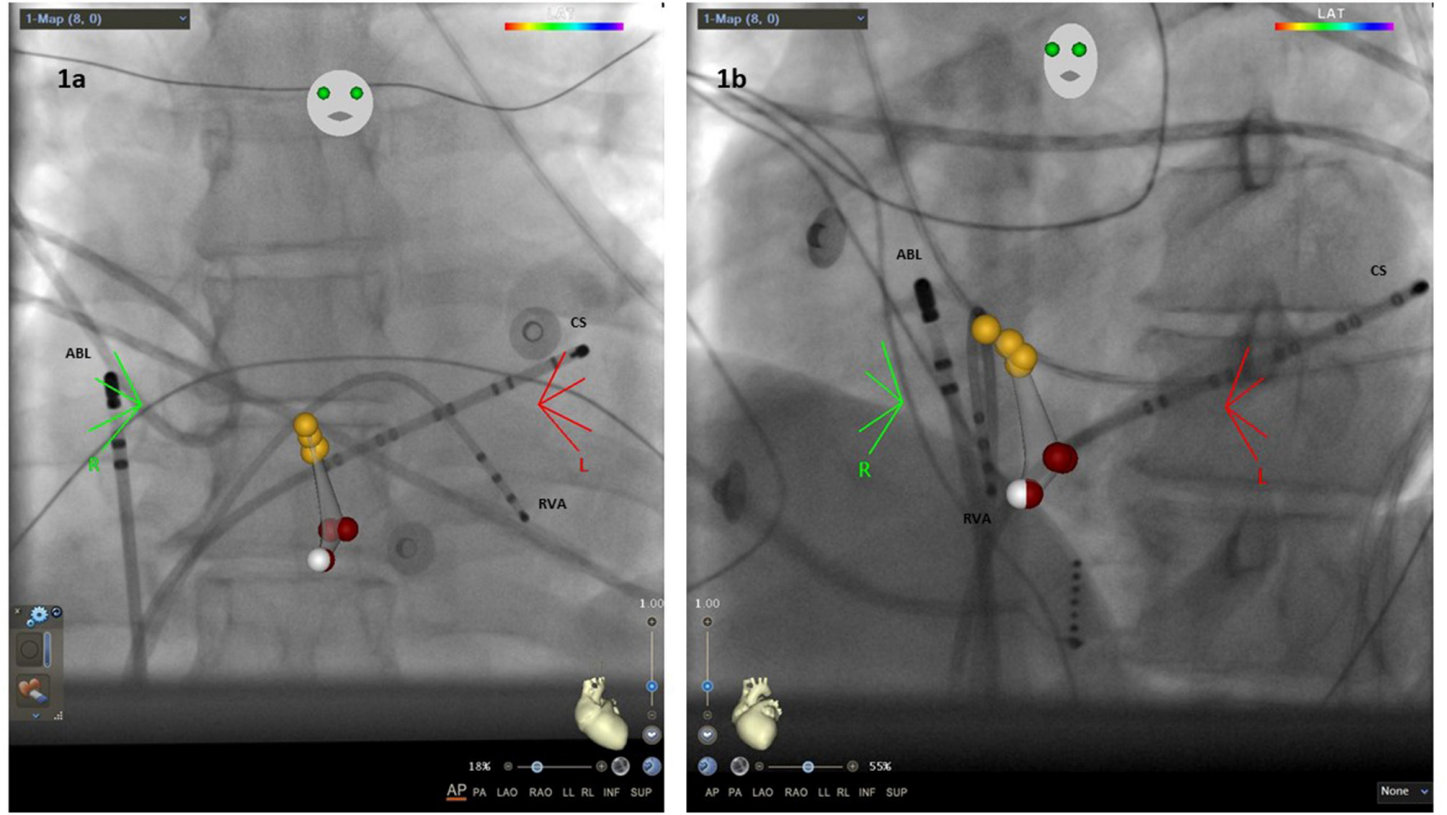
**(a)** Anterior-posterior (AP) view with the ablation catheter (ABL) placed in the right atrium, one catheter in the coronary sinus (CS) and one in the right ventricular apex (RVA). Yellow dots, region of HIS bundle; white dots, slow pathway region; red dots, ablation points. **(b)** Left anterior oblique (LAO) 40° view.

In ablations using EMS/FIS two diagnostic catheters (Biosense Webster) were placed in the RVA and CS. After diagnosis of AVNRT the ablation catheter (Navistar, Biosense Webster, d- or f-curve) was inserted. Afterwards, the FIS was initialized and fluoroscopic images were taken in anterior-posterior (AP) and left anterior oblique position (LAO, Figures 1a,b). Then a fast anatomical map (FAM) was performed to identify the HIS region and the region of ablation. As noted above, ablation sites were identified anatomically and by electrophysiology signals.

After ablation the stimulation maneuvers were repeated as described to determine the AV node conduction physiology. As previously described, the slow-pathway (SP) was defined as modified when an AH jump plus no more than one echo beat was observed but AVNRT was not inducible anymore. In contrast, SP elimination was defined as the absence of dual AV nodal physiology after ablation.

After successful ablation, pericardial effusion was excluded by echocardiography, patients were monitored till the next day and then discharged after another ECG had been performed and analyzed.

All authors had full access to the data, and have read and agreed to the manuscript as written. The study was approved by the local Institutional Review Board (Ärztekammer Nordrhein 69/2021) and conforms with the principles of the Declaration of Helsinki.

### Statistical Analysis

For global test statistics we used a significance level of <5% (*p* < 0.05). Continuous data is shown as mean ± standard deviation (SD). The Fisher's exact test and the Mann-Whitney-*U*-Test were used when appropriate. Data analysis was performed using IBM SPSS (IBM Corp, Armonk, NY, USA).

## Results

### General Aspects

All 119 consecutive patients were treated in 2018 and 2019. We analyzed the last 89 conventionally ablated patients [EMS/FIS(–)] and the first 30 patients ablated using EMS + FIS [EMS/FIS(+)]which was previously mainly used in complex ablations like atrial fibrillation, ventricular tachycardia or ectopic atrial tachycardia in our electrophysiology laboratory. All patients were successfully ablated without major complications. The patients' characteristics are shown in Table 1. The two groups showed no significant differences concerning age (51.01 ± 18.38 vs. 53.37 ± 17.07 years, *p* = 0.538), body mass index (BMI, 25.54 ± 4.83 vs. 24.11 ± 2.89, *p* = 0.130), ejection fraction (EF, 59.39 ± 2.99 vs. 58.33 ± 8.23, *p* = 0.303), presence of arterial hypertension (25.84 vs. 16.66%, *p* = 0.310) and chronic obstructive pulmonary disease (COPD, 7.8 vs. 3.33%, *p* = 0.396). In the EMS/FIS(–) group there was a trend toward more coronary artery disease (15.73 vs. 3.33%, *p* = 0.078) and diabetes mellitus II (11.23 vs. 0%, *p* = 0.056). There were significantly more female patients in the EMS/FIS(+) group (90 vs. 71.91%, *p* = 0.044).

**Table 1:** Baseline characteristics of the two groups EMS/FIS(–) and EMS/FIS(+).

	**EMS/FIS(–), *n* = 89**	**EMS/FIS(+), *n* = 30**	***p*-value**
Sex (female)	75 (71.91%)	27 (90%)	0.044
Age (years)	51.01 ± 18.39	53.37 ± 17.07	0.538
Body mass index	25.54 ± 4.83	24.11 ± 2.89	0.130
Ejection fraction (%)	59.39 ± 2.99	58.33 ± 8.23	0.303
Coronary artery disease	14 (15.73%)	1 (3.33%)	0.078
Diabetes mellitus II	10 (11.23%)	0 (0%)	0.056
Arterial hypertension	23 (25.84%)	5 (16.66%)	0.310
COPD	7 (7.8%)	1 (3.33%)	0.396
Procedure time (min)	66.55 ± 13.3	67.33 ± 13.81	0.783
Fluoroscopy time (sec)	449.80 ± 217.21	136.93 ± 109.28	<0.001
Dose area product (μGym^2^)	268.27 ± 265.20	41.07 ± 27.89	<0.001
Complications	1 (1.1%)	0 (0%)	0.564

*COPD, chronic obstructive pulmonary disease; FIS, fluoroscopy integrating system*.

### Learning Curve, PT, FT, and DAP

**Figure 2:**
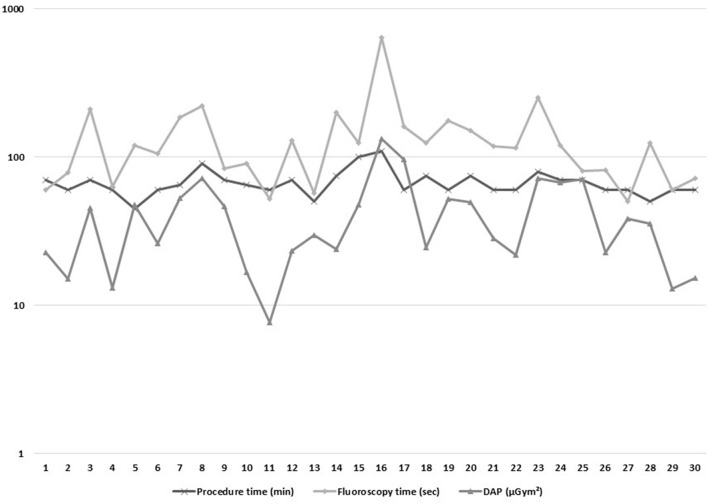
Characteristics of the 30 ablations using EMS/FIS with regard to procedure time (min), fluoroscopy time (sec), and dose-are-product (DAP, μGym^2^).

PT, FT, and DAP are shown for the 30 EMS/FIS (+) patients in Figure 2 (logarithmic scale). EMS/FIS had been established in our electrophysiology laboratory before and has been used in complex arrhythmias. Therefore, we did not observe a learning curve but stable procedure data since the first AVNRT procedure. Single cases with elevated FT and concomitant elevated DAP were mostly due to difficulties in placing the diagnostic catheters, especially the CS catheter.

**Figure 3:**
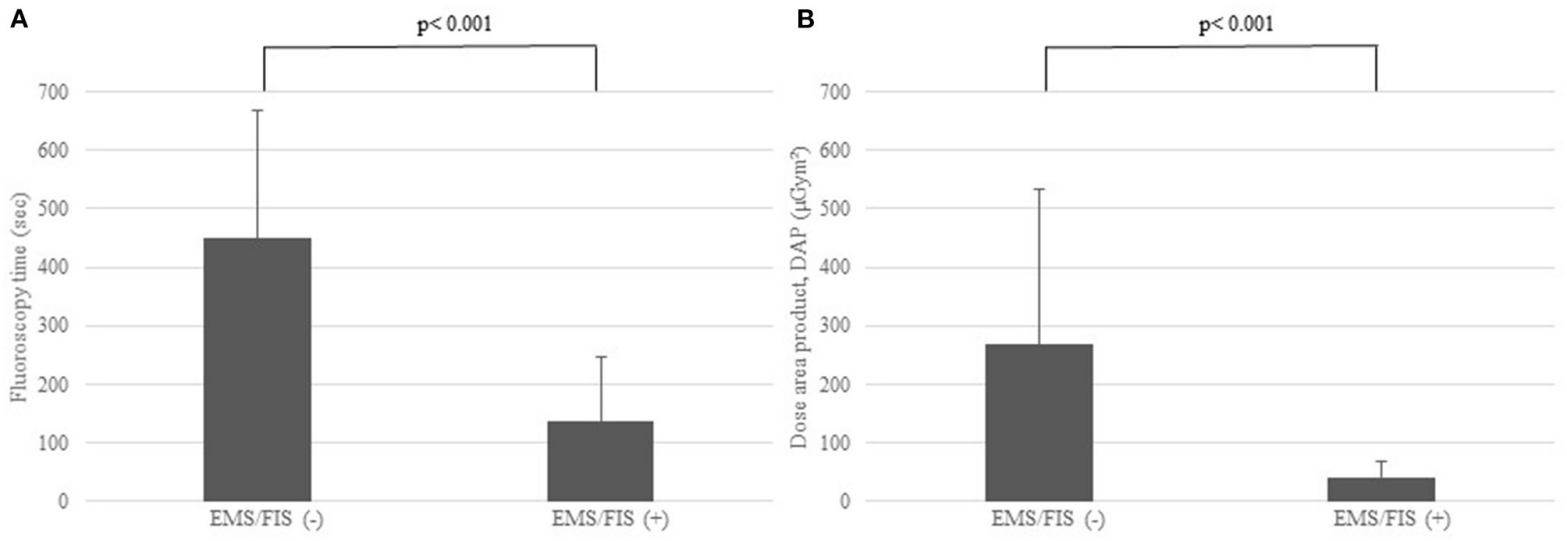
**(A)** Significant reduction of fluoroscopy time (FT, sec) in the EMS/FIS(+) vs. the EMS/FIS(–) group. **(B)** Significant reduction of dose-area-product (DAP, μGym^2^) in the EMS/FIS (+) vs. the EMS/FIS(–) group.

Comparing the EMS/FIS(–) and the EMS/FIS(+) group we found no significant differences with regard to PT (66.55 ± 13.3 vs. 67.33 ± 13.81 min, *p* = 0.783). PT was measured from groin puncture till removal of all sheaths. As for radiation data we found a highly significant reduction of FT (449.90 ± 217.21 vs. 136.93 ± 109.28 s, *p* < 0.001) and DAP (268.27 ± 265.20 vs. 41.07 ± 27.89 μGym^2^, *p* < 0.001) as shown in Figures 3A,B.

### AV Node Properties Post-ablation and Complications

After ablation during repeat stimulation maneuvers in the EMS/FIS(–) group a SP elimination was observed in 49.43% and a SP modulation in 50.57%. In the EMS/FIS(+) group, SP was eliminated in 46.67% and modified in 53.33% (*p* = 0.795).

Overall, one minor complication occurred. A female patient in the EMS/FIS(–) group suffered from a pseudoaneurysm after the procedure possibly as the result of an inadvertent arterial puncture. There was no need for surgical intervention.

## Discussion

Our study shows that the use of EMS+FIS in AVNRT ablation leads to a significant reduction of both FT and DAP without affecting the PT or the occurrence of complications. Furthermore, as the EMS/FIS is well-established in our clinic no learning curve was observed. We now use this approach as standard procedure in AVNRT ablations.

There is an increasing number of electrophysiologic studies (EPS) and ablations leading to a rising exposure to radiation for operators, staff, and patients. Radiation may lead to the development of chromosomal damage and malignant diseases (14). Several studies report on brain tumors in interventional cardiologists (15). During their work-life many operators develop cataracts (16). In summary, radiation should be kept to a minimum according to the ALARA principle (as low as reasonably achievable). The development of EMS has led to a significant decrease of radiation exposure in ablation procedures. It is mainly used in complex arrhythmias. EMS allow the operator to create a 3D map and to move the catheter in this map without fluoroscopy. The benefit and safety of EMS has widely been described as well as its potential in reducing FT (17–19). With the introduction of the FIS, the operator is able to integrate fluoroscopy images and cines leading to safer movement within the heart with respect to the anatomy and surrounding structures. Several studies showed benefit from using EMS + FIS compared to EMS alone (11, 12).

Many groups focus on the establishment of zero fluoroscopy ablation protocols (20). Pani et al. reported in their multicenter study on 430 patients ablated with the use of an EMS leading to a significant reduction of fluoroscopy and even zero fluoroscopy procedures after a short learning curve in the ablation of supraventricular tachycardias (21). Bhaskaran et al. reported on their experiences with an EMS in 30 patients and showed a safe ablation distance to His bundle region of >25 mm (22). Yamamoto et al. reported on the implementation of a predictive ablation point in an EMS resulting in simplification and improvement of their ablation procedures (23). In our study we found a significant reduction of FT and DAP using EMS/FIS. Remaining radiation time was mostly due to placement of the catheters in RVA and CS with increased radiation time in difficult anatomical circumstances. After initialization of the FIS no further radiation was needed in nearly all cases.

Despite the use of fluoroscopy saving technology, radiation should always be kept to a minimum. We try to keep the detector as close as possible to the patient, collimate as much as possible and reduce the frames per seconds to <3 whenever possible. Furthermore, fluoroscopy should be stored instead of cines and one should try to avoid extensive LAO angulation because the entrance site of the beam has a shorter distance to the operator than during RAO fluoroscopy as described by Heidbuchel et al. (9). The same author reported that fluoroscopy in LAO projections may expose the operator to six times more radiation than fluoroscopy in RAO projections.

Performing EPS with ablation targeting the SP is a well-established and safe therapy which is superior to antiarrhythmic drug therapy leading to an increase in the patient‘s quality of life (5, 24, 25). The most feared complication during AVNRT ablation is the development of permanent total AV block with the necessity to implant a permanent pacemaker. The incidence is rather rare (<1%) but transient AV block may occur more often (2%) (4, 26). It is known that the incidence of AV block is significantly higher when targeting the fast pathway so the slow-pathway is usually the ablation target (27). In our study we found, in both groups, no transient nor permanent AV block. Furthermore, we found no other complications beside one pseudoaneurysm in the conventional group without need for surgical treatment.

Concerning the endpoint of EPS in AVRNT ablation, several authors reported on the success rates and recurrence rates comparing SP ablation vs. SP modification, the latter meaning persistent dual AV node physiology after ablation. Clague et al. reported on 379 consecutive patients and summarized that SP modification was associated with a similar outcome compared to SP ablation (4). In another study Katritsis et al. reported on 1,007 patients with a success rate of almost 98% and a very low incidence of any AV block (<0.1%). They also observed that remaining dual AV node physiology was not correlated with a higher recurrence rate (28). In our study 44 patients (49.44%) in the EMS/FIS(–) and 14 patients (46.67%) in the EMS/FIS(+) had SP ablation, the others had SP modification (*p* = 0.795). Of note, five patients presented with recurrent AVNRT after initial ablation in our clinic or elsewhere. In all these cases, during initial ablation remaining evidence of dual AV nodal physiology was found. In a recent publication Wegner et al reported on a long-time follow-up of more than 3,000 patients after AVNRT ablation. They showed that recurrence of AVNRT is very rare and found that absence of junctional beats during ablation and the occurrence of other supraventricular arrhythmias were predictors for the recurrence of AVNRT (29).

## Limitations

The study has several relevant limitations due to its retrospective and monocentric, non-randomized character. Unfortunately, data concerning the number and duration of RF applications as well as potential changes in HV intervals were not collected; therefore, we are unable to compare them in detail, however in our experience they did not differ significantly. Furthermore, the number of patients is rather small. Nevertheless, we think the number of patients is adequate to show the significant reduction of both DAP and FT using the EMS/FIS system.

## Conclusion

Ablation of AVNRT using an EMS/FIS system is safe and feasible and leads to a significant reduction of both DAP and FT without affecting the PT and safety. Reduction of radiation exposure is beneficial not only for the patient but also for the operator and the lab staff.

## Data Availability Statement

The original contributions presented in the study are included in the article/supplementary material, further inquiries can be directed to the corresponding author/s.

## Ethics Statement

The studies involving human participants were reviewed and approved by Ärztekammer Nordrhein, 69/2021. Written informed consent for participation was not required for this study in accordance with the national legislation and the institutional requirements.

## Author Contributions

CB and J-EG: design, data collection, and writing. AB: analysis and review. MS: analysis and supervision. BK: design and statistical supervision. D-IS: supervision and review. All authors contributed to the article and approved the submitted version.

## Conflict of Interest

The authors declare that the research was conducted in the absence of any commercial or financial relationships that could be construed as a potential conflict of interest.

## Publisher's Note

All claims expressed in this article are solely those of the authors and do not necessarily represent those of their affiliated organizations, or those of the publisher, the editors and the reviewers. Any product that may be evaluated in this article, or claim that may be made by its manufacturer, is not guaranteed or endorsed by the publisher.
